# Intrinsic Plasma Cell Differentiation Defects in B Cell Expansion with NF-κB and T Cell Anergy Patient B Cells

**DOI:** 10.3389/fimmu.2017.00913

**Published:** 2017-08-02

**Authors:** Swadhinya Arjunaraja, Brent D. Nosé, Gauthaman Sukumar, Nathaniel M. Lott, Clifton L. Dalgard, Andrew L. Snow

**Affiliations:** ^1^Department of Pharmacology and Molecular Therapeutics, Uniformed Services University of the Health Sciences, Bethesda, MD, United States; ^2^Department of Anatomy, Physiology and Genetics, Uniformed Services University of the Health Sciences, Bethesda, MD, United States; ^3^The American Genome Center, Uniformed Services University of the Health Sciences, Bethesda, MD, United States

**Keywords:** B cell Expansion with NF-κB and T cell Anergy, CARD11, plasma cells, BLIMP-1, antibodies, humans, primary immunodeficiency

## Abstract

B cell Expansion with NF-κB and T cell Anergy (BENTA) disease is a novel B cell lymphoproliferative disorder caused by germline, gain-of-function mutations in the lymphocyte scaffolding protein CARD11, which drives constitutive NF-κB signaling. Despite dramatic polyclonal expansion of naive and immature B cells, BENTA patients also present with signs of primary immunodeficiency, including markedly reduced percentages of class-switched/memory B cells and poor humoral responses to certain vaccines. Using purified naive B cells from our BENTA patient cohort, here we show that BENTA B cells exhibit intrinsic defects in B cell differentiation. Despite a profound *in vitro* survival advantage relative to normal donor B cells, BENTA patient B cells were severely impaired in their ability to differentiate into short-lived IgD^lo^CD38^hi^ plasmablasts or CD138^+^ long-lived plasma cells in response to various stimuli. These defects corresponded with diminished IgG antibody production and correlated with poor induction of specific genes required for plasma cell commitment. These findings provide important mechanistic clues that help explain both B cell lymphocytosis and humoral immunodeficiency in BENTA disease.

## Introduction

Our group recently characterized a novel congenital B cell-specific lymphoproliferative human disorder termed as B cell Expansion with NF-κB and T cell Anergy (BENTA) disease (1, 2). BENTA disease is caused by heterozygous gain-of function (GOF) mutations in the gene *CARD11*. *CARD11* encodes a lymphocyte-restricted scaffold protein (also known as CARMA1) that bridges antigen receptor (AgR) engagement with various downstream signaling pathways, such as c-Jun N-terminal kinase (JNK), mechanistic target of rapamycin (mTOR), and most notably, the canonical NF-κB pathway (3–5). The NF-κB family of transcription factors governs the expression of multiple genes involved in immune cell survival, proliferation and effector functions (6). In resting lymphocytes, the inhibitory “linker” domain of CARD11 maintains the protein in a closed, inactive conformation, preventing interaction with other proteins. Upon AgR ligation, the phosphorylation of several serines in the linker allows CARD11 to multimerize and recruits BCL10 and MALT1 to form the “CBM (CARD11-BCL10-MALT1) complex” (7, 8). This signalosome triggers a complex, dynamic series of signaling events that eventually culminates in the activation of the inhibitor of κB kinase (IKK) complex (9–11). Active IKK in turn phosphorylates the inhibitor of κB (IκB), marking it for ubiquitination and proteasomal degradation, and thereby promotes the nuclear translocation of NF-κB (p65:p50 heterodimers) for gene transcription. Because NF-κB-driven gene transcription is critical for protective innate and adaptive immunity, disruptive mutations in many components of this canonical signaling pathway often manifest in distinctive human primary immunodeficiencies (PIDs) (12–15). Certain genetic defects, such as *NFKB1* haploinsufficiency, can give rise to both PID and lymphoproliferative disease (16).

Sixteen patients have been definitively diagnosed with BENTA disease to date, based on the detection of five distinct, heterozygous germline GOF *CARD11* mutations. Most involve single missense mutations (E134G, G123S, G123D, and C49Y) (2, 17, 18), except in one family carrying an additional four amino acid deletion in the coiled-coil (CC) domain (unpublished data). Similar to somatic *CARD11* GOF mutations found in diffuse large B cell lymphomas (DLBCL) and other lymphoid malignancies, germline GOF mutations in BENTA reside in the N-terminal portion of *CARD11* encoding the CARD, LATCH, and CC domains (19, 20). These GOF mutations abrogate the requirement for AgR-induced phosphorylation of the linker domain by disrupting autoinhibition afforded by several repressive elements, eliciting an “open” conformation for unimpeded BCL10/MALT1 recruitment and constitutive activation of NF-κB (21, 22). Indeed, primary B and T cells from BENTA patients demonstrate evidence of spontaneous CARD11 aggregation and elevated NF-κB signaling. Similarly, ectopically expressed BENTA-associated CARD11 mutants spontaneously assemble into large protein aggregates containing MALT1, BCL10 and phosphorylated IKKα/β in transfected B and T cell lines, inducing constitutive NF-κB activation without AgR stimulation (2). These GOF *CARD11* mutations may predispose BENTA patients to lymphoid malignancy later in life, as B cell clones acquire additional mutations over time. Indeed, at least two patients reported B cell neoplasms in adulthood (2) (unpublished data).

Polyclonal B cell lymphocytosis noted in early childhood is a fundamental diagnostic feature of BENTA disease, accompanied by splenomegaly. Immunologic phenotyping reveals excessive accumulation of both immature CD10^+^ CD24^hi^ CD38^hi^ transitional B cells and polyclonal IgD^hi^ mature naive B cells in the blood, generally with normal numbers of T cells. Nevertheless, autoimmune disease symptoms are not commonly seen in these patients, with few autoantibodies detected. Furthermore, BENTA patients display several hallmarks of PID. Frequent sinopulmonary and ear infections are common in all patients, and opportunistic viral infections such as chronic Epstein-Barr virus (EBV), BK virus, and molluscum contagiosum are noted in some patients. Most patients exhibit poor humoral immune responses to T cell-independent pneumococcal and meningococcal polysaccharide-based vaccines. Low antibody titers to some T cell-dependent vaccines such as Varicella Zoster virus (VZV) and measles are also observed in some patients. Impaired humoral immunity is also showcased by extremely low frequencies of circulating class-switched as well as CD27^+^ memory B cells. Several patients also have low serum IgM, with fluctuating IgA and IgG levels that often fall in the lower end of normal range.

In this study, we aimed to determine why antibody responses may be suboptimal in BENTA patients, focusing on the B cells themselves. Compared to healthy control B cells, our data demonstrate that BENTA B cells exhibit severely impaired differentiation into short-lived IgD^lo^ CD38^hi^ and long-lived CD38^hi^ CD138^hi^ plasma cells (PCs) *in vitro*, despite normal proliferation and enhanced survival after polyclonal stimulation. Consistent with these differentiation defects, BENTA B cells exhibited low IgG secretion and attenuated induction of key differentiation factors that govern the distinct transcriptional program associated with PC commitment. These results suggest that activated BENTA B cells have profound, intrinsic differentiation defects that compromise normal humoral immunity.

## Materials and Methods

### Naive B Cell Isolation

B cell Expansion with NF-κB and T cell Anergy patient blood samples were obtained after written informed consent through protocols approved by the institutional review boards of the National Institutes of Health (NIH) and USUHS. Anonymous buffy coat samples from healthy normal donors were kindly provided by the NIH Blood Bank. Peripheral blood mononuclear cells (PBMCs) were isolated using Ficoll-paque plus (GE Healthcare) density gradient centrifugation as previously described (17). ACK lysis buffer [5 min at room temperature (RT)] was used to lyse erythrocytes. Naive B cells were subsequently isolated by negative selection using an EasySep immunomagnetic bead sorting kit (StemCell Technologies, Cat # 19254). CD10^+^ transitional B cells were simultaneously depleted using the EasySep human CD10 positive selection kit (StemCell Technologies, Cat # 18358).

### Activation of Human Naive B Cells *In Vitro*

Naive B cells from healthy donors and BENTA patients were cultured in Iscove’s modified Dulbecco medium (IMDM) plus 10% fetal bovine serum supplemented with penicillin/streptomycin (Sigma). Purified B cells were plated at 1 × 10^6^ cells/mL in 24-well flat-bottom plates (1 mL/well) or 96-well round bottom plates (0.2 mL/well). Cells were activated using (a) 100 ng/mL of dextran-conjugated anti-IgD antibodies (αIgD-DEX) (Fina Biosolutions), (b) SAC (Protein A from Staphylococcus *aureus* Cowan I) + 200 U/mL of rIL-2 (Peprotech), or (c) 10 µg/mL of Affinipure F(ab′_2_) fragment-specific goat anti-human IgM (Jackson Immunoresearch Laboratories) plus 1 µg/mL anti-CD40 Ab (AF632, R&D Biosystems) plus IL-21 (100 ng/mL, Biolegend) and IL-2 (20 U/mL, Biolegend). For (c), additional combinations of cytokines (Biolegend) IL-4 (100 ng/mL) + IL-13 (50 U/mL), or IL-10 (25 ng/mL) were concomitantly used to induce isotype-specific antibody responses. For long-lived plasma cell generation, IL-6 (10 ng/mL), IFN-α (100 U/mL), and IL-21 (100 ng/mL) along with Lipid Mixture 1, chemically defined (Sigma, 1:500 dilution) and MEM amino acids solution (Sigma, 1:100 dilution) were added on day 6 to cells that were previously stimulated with α-IgM Ab/α-CD40 Ab/IL-21/IL-2. Culture media was changed every 4 days.

### Proliferation Assays

Naive B cells were plated at a concentration of 1 × 10^6^ cells/mL in 96-well plates (200 μL/well) and activated using different stimuli as specified previously. Four days later, proliferation (i.e., DNA replication) was measured using Click-iT EdU AF-488 flow cytometry assay kits (Life Technologies, C-10420). To track cell division, naive B cells were labeled with PBS containing 1 µM carboxyfluorescein succinyl ester (CFSE, Invitrogen), washed 2× in complete RPMI, and stimulated with anti-IgM/anti-CD40/IL-21/IL-2. Cells were harvested on days 4–6, stained with TO-PRO-3 to discriminate live/dead cells, and analyzed by flow cytometry. Data analysis was performed using FlowJo software (FlowJo LLC). Proliferation index was calculated as total # of divisions/total # of dividing cells.

### ELISA

Supernatants from activated and resting B cell cultures were taken on day 10 and quantified by ELISA. Immulon 2 plates were coated with 100 μL/well of goat anti-human IgM (μ chain specific) and IgG (H + L) (Southern Biotech) at a concentration of 1 µg/mL and incubated overnight at 4°C. The plates were then washed twice using wash buffer (PBS + 0.05% Tween 20) and blocked with 100 μL/well of PBS plus 1% BSA for 2 h at 37°C. Human IgM whole molecule (Pierce) and human IgG isotype control (Southern Biotech) were used as standards starting from 1 µg/mL in the first well and serially diluted 1:2. Cell supernatants at 1:2 dilution were added in triplicate to plates, then incubated at 37°C for 2 h and then washed twice with wash buffer. Goat anti-human IgM (Fc5μ fragment specific) or goat anti-human IgG (H + L) conjugated to horseradish peroxidase (HRP) from Pierce was added at 1:50,000 dilution (100 μL/well) and incubated at 37°C for 1 h. Plates were again washed twice and then incubated with 100 μL/well of TMB substrate (Thermo Scientific) at RT for 10–15 min. Sulfuric acid (2 N, 100 µL/well) was added to stop the TMB reaction. Absorbance was read at 450 nm using a Biotek Synergy H1 hybrid microplate reader. IgG isotypes from B cell culture supernatants were quantified using Ready-Set-Go Human IgG1, IgG3, and IgG4 ELISA kits from eBiosciences according to the manufacturer’s instructions.

### Enzyme-Linked Immunosorbent Spot (ELISPOT) Assay

Enzyme-linked immunosorbent spot assays were performed using the CTL Human IgM/IgG double-color ELISPOT assay kit (Cellular Technology Limited, Cat # hIgMIgG-DCE-2M/2). ELISPOT plates were coated overnight using human Ig capture Ab. The plates were then washed with 200 μL/well of sterile PBS at RT. B cells cultured in complete IMDM were activated with various stimuli and harvested on day 4. Cells were washed three times with CTL-serum free media and plated in ELISPOT plates, starting at 25,000 cells/well with a twofold serial dilution, and subsequently incubated for 16–24 h at 37°C. Plates were then washed twice with PBS and twice with 0.05% Tween-PBS. The IgM/IgG detection solution was then added and incubated at RT for at least 2 h. Plates were washed twice with 0.05% Tween-PBS and incubated with the tertiary solution at RT for 1 h. Plates were washed again with 0.05% Tween-PBS and developed using blue or red developer solution provided in the kit to detect IgG or IgM secreting cells, respectively. Detection, tertiary, and developer solutions were prepared according to the manufacturer’s instructions.

### Antibody Staining and Flow Cytometry

On day 6 on *in vitro* culture with anti-IgM/anti-CD40 Ab/IL-21/IL-2, activated B cells were collected by centrifugation (~400 × *g* for 5 min) and resuspended in FACS buffer (PBS + 1% FBS + 0.01% sodium azide). Anti-human IgD-PE and anti-human CD38-FITC antibodies (5 µL each) were added and mixed by vortexing, then incubated on ice for 30 min. The cells were then washed with FACS buffer and centrifuged as above. Buffer was decanted, and cell pellets were resuspended in 0.4 mL FACS buffer and collected on an Accuri C6 flow cytometer (Becton Dickinson). To quantify long-lived plasma cells in culture, activated B cells were collected on day 13 and stained with anti-human IgD-PE, anti-human CD38-FITC, and anti-human CD138-APC (5 µL each) in FACS buffer. For surface IgG expression, cells on day 10 were stained with anti-IgG-APC (Biolegend). Cells were washed and analyzed on the Accuri C6 as described above. Data analysis was performed using FlowJo software (FlowJo LLC).

### RNA Isolation and Quantitative PCR

Total RNA was extracted from anti-IgM/anti-CD40/IL-21/IL-2 activated (day 4) control and BENTA patient B cells using Qiagen RNeasy Mini plus kit (Cat # 74134). cDNA conversion was performed using iScript Advanced cDNA synthesis kit (Biorad). Primers for qPCR were designed using Primer-Blast (https://www.ncbi.nlm.nih.gov/tools/primer-blast/) and synthesized from Integrated DNA Technologies and Biomedical Instrumentation Center at USUHS, Bethesda. qPCR was performed on a CFX384 Real-Time PCR Detection System (Bio-Rad) using SSOAdvanced Universal SYBR Green master mix (BioRad) as per manufacturer’s instructions. Data were normalized based on expression of two housekeeping genes (*HPRT2* and *RPL13*).

### Transcriptome Profiling by RNA-Seq

As described previously (17), RNA integrity was assessed using automated capillary electrophoresis on a Fragment Analyzer (Advanced Analytical). Total RNA input of 75 ng was used for library preparation using the Neoprep Stranded mRNA Library Preparation Kit (Illumina, San Diego, CA, USA). Sequencing libraries were quantified by PCR using KAPA Library Quantification Kit for NGS (Kapa, Wilmington, MA, USA) and assessed for size distribution on a Fragment Analyzer. Sequencing libraries were pooled and sequenced on a NextSeq 500 Desktop Sequencer (Illumina) using NextSeq 500 High Output Kit v2 with paired-end reads at 75 bp length. Raw sequencing data were demuxed using bcl2fastq2 conversion software 2.17 before alignment using TopHat Alignment v1.0. Differential transcript expression analysis was performed using Cufflinks Assembly & DE v1.1.0 on BaseSpace Onsite (Illumina). Heatmaps of differentially expressed genes were generated using ClustVis software. Data are deposited in NCBI’s Gene Expression Omnibus (GEO Series accession number GSE101756).

### Immunoblotting

Activated naive B cells (~1 × 10^6^ cells) from healthy controls and BENTA patients stimulated with anti-IgM/anti-CD40/IL-21/IL-2 were collected by centrifugation and resuspended in 1% NP-40 lysis buffer (50 mM Tris pH 7.4, 150 mM NaCl, 1% NP-40, 0.5% sodium dexycholate, 0.5 mM EDTA) supplemented with complete protease inhibitor mixture (Roche). Cell pellets were vortexed briefly and incubated in ice for 30 min. Insoluble material was removed by centrifugation (16,000 × *g* for 5 min). Protein concentration was determined by BCA assay (Thermo Fisher Scientific). Equal amounts of protein lysate were separated on 4–20% SDS-PAGE gradient gels (Bio-Rad) and transferred to nitrocellulose membrane. For IgM detection, day 10 cell supernatants (25 mL) were separated by SDS-PAGE and transferred as above. After blocking in 0.2% iBlok (Bio-Rad) +0.1% Tween 20, membranes were probed with the following antibodies: anti-XBP1s rabbit mAb (D2C1F), anti-BLIMP-1/PRDI-BF1 rabbit mAb (C14A4), anti-AID mouse mAb (L7E7), anti-IRF4 rabbit mAb, and anti-GAPDH rabbit mAb (Cell Signaling Technology); anti-human IgM (Rockland), or anti-human IgG (Jackson Immunoresearch Laboratories). Bound Abs were detected using HRP-conjugated secondary Abs (Southern Biotech) and enhanced chemiluminescence (Thermo Fisher Scientific), visualized on a ChemiDoc system (Bio-Rad). Spot densitometry analysis was performed using Image Lab software (Bio-Rad).

## Results

### Normal Proliferation of BENTA B Cells upon Activation

**Figure 1:**
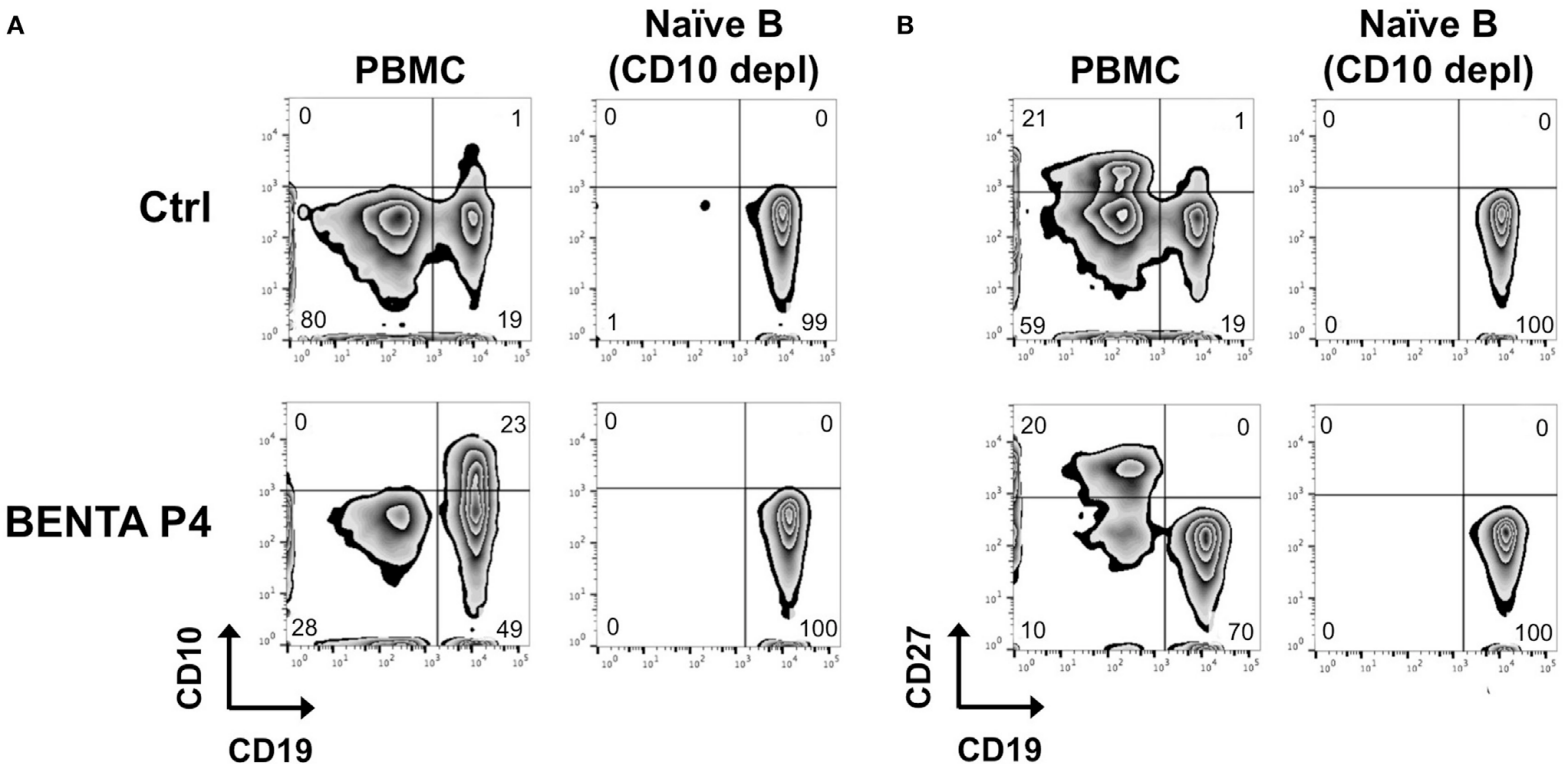
Naive B cell isolation from healthy donor and B cell Expansion with NF-κB and T cell Anergy (BENTA) patient peripheral blood mononuclear cell (PBMC). Flow cytometric analysis of CD19^+^ CD10^+^ transitional B cells **(A)** and CD19^+^ CD27^+^ memory B cells **(B)** from healthy human control (top row) versus BENTA patient P4 PBMC (bottom row) before and after naive B cell isolation along with CD10^+^ depletion.

Because BENTA patients are largely devoid of memory B cells (2), we purified naive B cells from BENTA patients (Table 1) and healthy control subjects for direct comparative studies. As shown previously, B cell specific lymphoproliferation was evident in PBMC isolated from BENTA patients (~50–80% CD19^+^, Figure 1), compared to healthy controls (~10–20% CD19^+^). In contrast to controls, we noted a significant proportion of CD10^+^ transitional B cells and virtually no CD27^+^ memory B cells in BENTA patients. Our customized immunomagnetic isolation of naive B cells efficiently removed CD10^+^ transitional B cells and CD27^+^ memory B cells from both BENTA patient and control samples (Figures 1A,B).

**Table 1:** B cell Expansion with NF-κB and T cell Anergy patients included in this study.

Patient	CARD11 mutation
P2	E134G
P3	E134G
P4	G123S
P6	G123D

*Heterozygous missense mutations in CARD11 are listed for each patient*.

**Figure 2:**
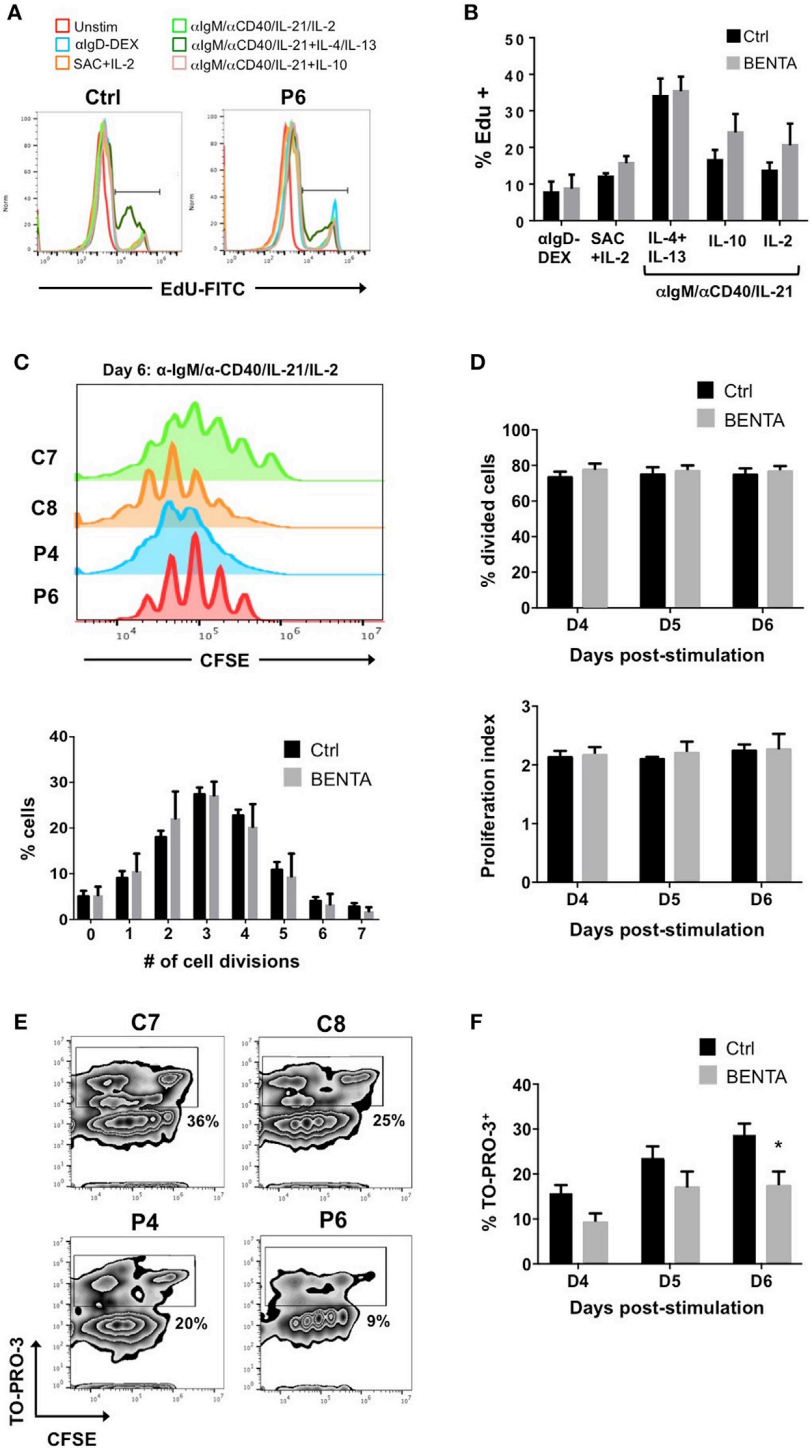
Normal proliferation of B cell Expansion with NF-κB and T cell Anergy (BENTA) B cells upon activation in culture. **(A)** *In vitro* proliferation assay using EdU incorporation for healthy control and BENTA patient P6 B cells in response to various activating stimuli in culture, assessed by flow cytometry. Data are representative of seven independent experiments. **(B)** Quantification of proliferating cells (% EdU+) for healthy control (black) and BENTA B cells (gray) subjected to different stimuli in culture. Data are mean ± SEM of seven independent experiments (*n* = 6 controls, four BENTA patients). **(C)** Flow cytometric CFSE dilution analysis of naive control (C7, C8) and BENTA patient (P4, P6) B cells activated with anti-IgM/anti-CD40/IL-21/IL-2 for 6 days. The percentage of control (black) and BENTA (gray) B cells that divided 0–7 times on day 6 is displayed below. Data are mean ± SEM for two independent experiments (*n* = 4 controls, three BENTA patients). **(D)** Bar graphs displaying the percentage of divided cells (top, mean ± SEM) and proliferative index (bottom, mean ± SEM) for data shown in **(C)**, days 4–6 in culture. **(E)** Flow cytometric analysis of CFSE dilution versus TO-PRO-3 live/dead cell indicator staining on day 6 of cultures in **(C)**. Percentage of dead cells (TO-PRO-3^+^) is noted for gates shown in each plot. **(F)** Quantification of dead (TO-PRO3^+^) control (black) and BENTA (gray) B cells over days 4–6 in culture. Data are mean ± SEM for two independent experiments (*n* = 4 controls, three BENTA patients). Asterisk denotes statistical significance (Student’s *t*-test with Welch correction, *p* < 0.05).

We first tested the proliferative capacity of naive BENTA patient B cells compared to control B cells in response to various stimuli *in vitro*. We first measured EdU incorporation on day 4. Neither unstimulated control nor BENTA B cells proliferated in culture after 4 days, confirming that resting patient B cells are not actively proliferating *in vitro*. Both control and BENTA B cells showed comparable EdU incorporation when stimulated with polyclonal dextran-conjugated anti-IgD (αIgD-Dex), a T cell-independent type-2 like stimulus, and heat killed *S. aureus* Cowan I plus IL-2 (SAC + IL-2), a polyclonal B cell mitogen (Figure 2A). Moreover, BENTA B cells exhibited robust proliferation in response to a T cell dependent type stimulus including B cell receptor (BCR) crosslinking with anti-IgM, CD40 ligation using anti-CD40 antibody, and IL-21, plus additional cytokines IL-2, IL-4 + IL-13, or IL-10. Although BENTA B cells showed a trend toward increased EdU incorporation for some stimuli, we found no significant differences in proliferation of activated control and BENTA B cells in response to any stimuli tested (Figure 2B). Similar results were obtained when we tracked cell division by CFSE dilution over 6 days in culture (Figures 2C,D). Control and BENTA B cell cultures showed comparable proliferation when stimulated with anti-IgM, anti-CD40, IL-21, and IL-2, as measured by the percentage of divided cells, the proliferation index (Figure 2D), as well as the percentage of cells within each round of division by day 6 (Figure 2C). However, activated BENTA B cells showed significantly less cell death in culture compared to controls, based on staining with the live/dead cell indicator TO-PRO-3 (Figures 2E,F). As we have noted previously (1, 17), these data hence suggest that BENTA patient B cells generally display normal proliferation and enhanced viability compared to control B cells upon polyclonal stimulation.

### Impaired Plasma Cell Differentiation of BENTA B Cells

**Figure 3:**
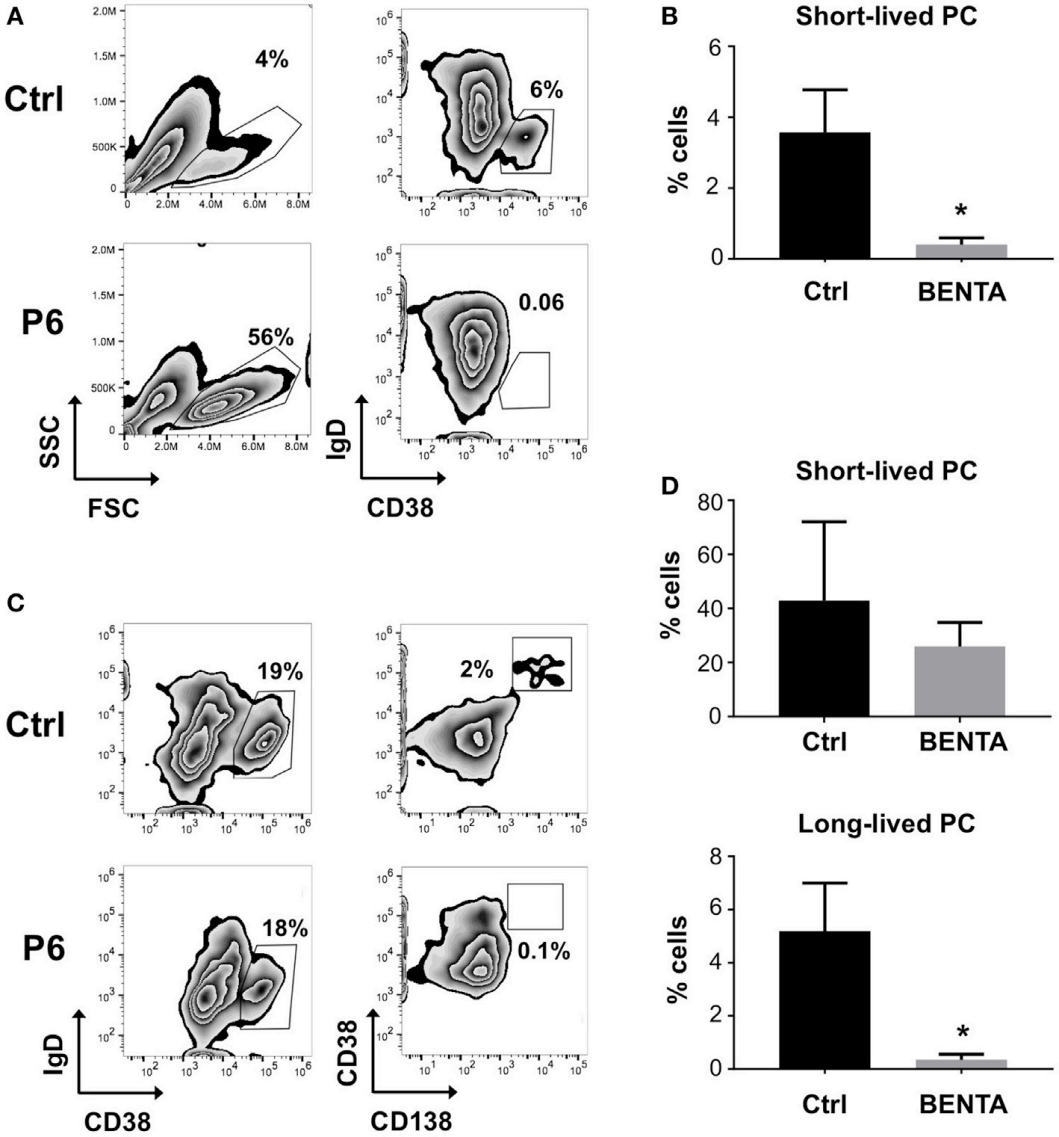
Impaired plasma cell differentiation of B cell Expansion with NF-κB and T cell Anergy (BENTA) B cells in culture. **(A)** Purified control and BENTA P6 naive B cells were stimulated with anti-IgM/anti-CD40/IL-21 and IL-2 for 6 days. The percentages of short-lived plasma cells (PCs) (IgD^lo^ CD38^hi^ cells) in control (upper panel) and BENTA (lower panel) B cells were identified by gating on viable cells based on forward/side scatter. Data are representative of seven independent experiments. **(B)** Bar graph representing percentages of short-lived PCs (mean ± SEM) in control (black) and BENTA B cell cultures (gray) from seven independent experiments (*n* = 6 controls, four BENTA patients). Asterisk denotes statistical significance (Student’s *t*-test with Welch correction, *p* < 0.05). **(C)** Purified control and BENTA P6 naive B cells stimulated in **(A)** were supplemented with additional cytokines (IFN-α, IL-6, and IL-21) from day 6 onward until day 13. The percentage of short-lived PC (IgD^lo^ CD38^hi^ cells, left) and long-lived PCs (CD38^hi^ CD138^hi^ cells, right) for control and BENTA B cells were determined by flow cytometry. **(D)** Bar graph representing percentages (mean ± SEM) of short-lived (top) and long-lived PC (bottom) in control (black) and BENTA (gray) B cell cultures from seven independent experiments (*n* = 6 controls, four patients). Asterisks denote statistical significance (Student’s *t*-test with Welch correction, *p* < 0.05).

Clinical data from previous reports indicate that BENTA patients have an unusually low number of class-switched and/or memory B cells *in vivo* and cannot mount lasting humoral responses to certain vaccines (2, 17, 18, 23). Therefore, we investigated whether BENTA B cells can differentiate into plasma cells (PCs) *in vitro*, based on previously published methodology (24–26). Naive control and BENTA B cells were activated with anti-IgM, anti-CD40, and IL-21 plus IL-2 to promote PC differentiation in culture. On day 6, activated B cells were collected and examined for short-lived PC differentiation, identified by CD38 upregulation and IgD downregulation. Forward/side scatter gating as shown confirmed more viable B cells in BENTA patients compared to controls, consistent with our previous report (Figure 3A) (17). Under these culture conditions, 3.6 ± 1.2% of control B cells were able to differentiate into IgD^lo^ CD38^hi^ short-lived PC by day 6 (Figures 3A,B). Conversely, BENTA B cells failed to differentiate into short-lived PC under these culture conditions (0.4 ± 0.2%, Figures 3A,B). To examine continued differentiation into long-lived CD138^+^ PC, we supplemented anti-IgM/anti-CD40/IL-21/IL-2 stimulated cultures on day 6 with IL-6, IFN-α, and IL-21, which further promotes robust STAT3 activation (26). These additional cytokines promoted the differentiation of both control and BENTA B cells into IgD^lo^ CD38^hi^ cells (short-lived PC) by day 13 (Figure 3D). However, while 5.2 ± 1.8% of control B cells differentiated into CD138^hi^ long-lived PC, BENTA B cells failed to differentiate into long-lived PC in this setting (0.34 ± 0.2%, Figures 3C,D). Although *in vitro* PC differentiation from human naive B cells is very inefficient, averaged data including four separate controls and 4 BENTA patients showed clear, statistically significant differences in PC populations across multiple experiments (Figures 3B,D). Collectively, our findings suggest that PC differentiation is impaired for BENTA B cells.

### Poor IgG Responses from Stimulated BENTA B Cells

**Figure 4:**
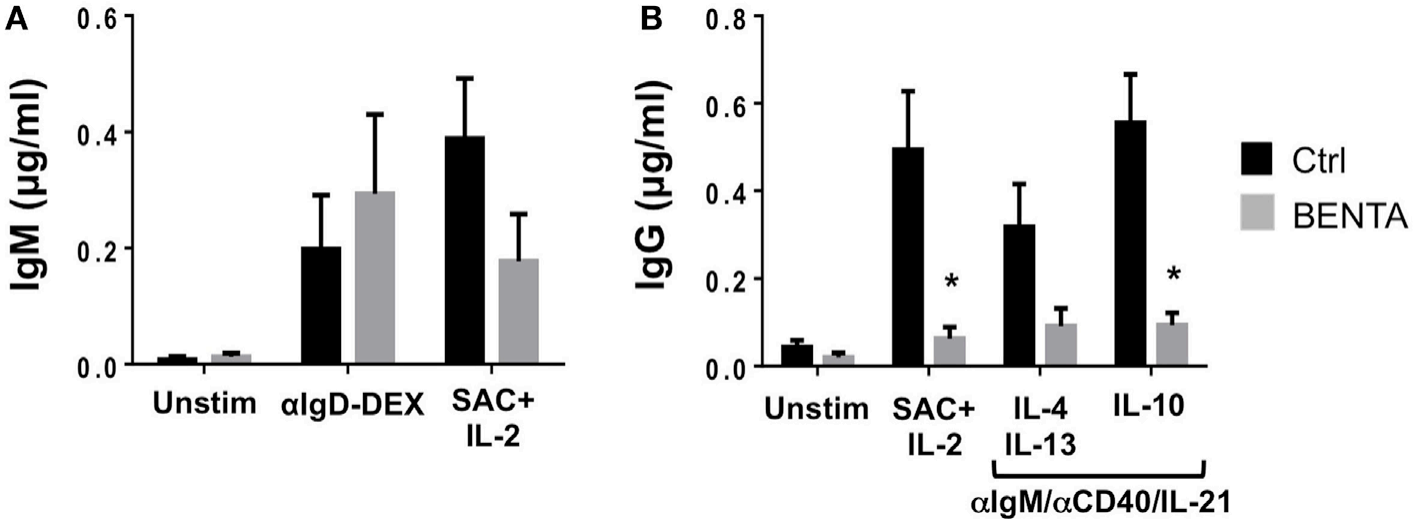
Poor IgG production from stimulated B cell Expansion with NF-κB and T cell Anergy (BENTA) B cells. **(A,B)** Supernatants from purified healthy control (black) versus BENTA (gray) naive B cells cultured with various activating stimuli were collected on day 10 and used for quantification of IgM **(A)** or IgG **(B)** by ELISA. Data represent IgM or IgG levels (mean ± SEM) for control (*n* = 6) and BENTA patient (*n* = 4) B cells from seven independent experiments. Asterisks denote statistical significance (Student’s *t*-test with Welch correction, *p* < 0.05).

Given this impairment in PC differentiation, we further examined *in vitro* antibody responses from control and BENTA patient naive B cells. Supernatants were collected from stimulated B cell cultures on day 10 and evaluated for IgM and IgG antibody responses by ELISA. Unstimulated control and BENTA B cells produced minimal IgM or IgG antibodies (Figures 4A,B). Both control and BENTA B cells produced substantial amounts of IgM in response to stimulation with αIgD-Dex and SAC + IL-2 stimulation (Figure 4A). Although we observed a trend toward decreased IgM production with SAC + IL-2 in BENTA B cells, as noted in previous studies, this difference was not statistically significant. As expected, αIgD-DEX alone, a T cell-independent type-2 like stimulus, did not induce detectable levels of IgG responses in either group (data not shown). Control and BENTA B cells given T cell-dependent stimuli (anti-IgM or anti-IgD-DEX plus anti-CD40/IL-21/IL-2) with or without additional cytokines also produced IgM (Figure S1 in Supplementary Material). However, BENTA B cells activated with SAC + IL-2 or anti-IgM/anti-CD40/IL-21 (±additional cytokines) produced significantly lower amounts of IgG in culture compared to control B cells (Figure 4B). Consistent with poor IgG secretion overall, we also noted less production of IgG isotypes such as IgG1, IgG3, and IgG4 in response to type 1 (anti-IgM/anti-CD40/IL-21 + IL-10) or type 2 (anti-IgM/anti-CD40/IL-21 + IL-4 + IL-13) specific stimuli in BENTA B cell culture supernatants (Figure S2 in Supplementary Material). Taken together, these findings suggest that activated BENTA B cells elicit poor IgG responses *in vitro* despite relatively normal IgM responses.

**Figure 5:**
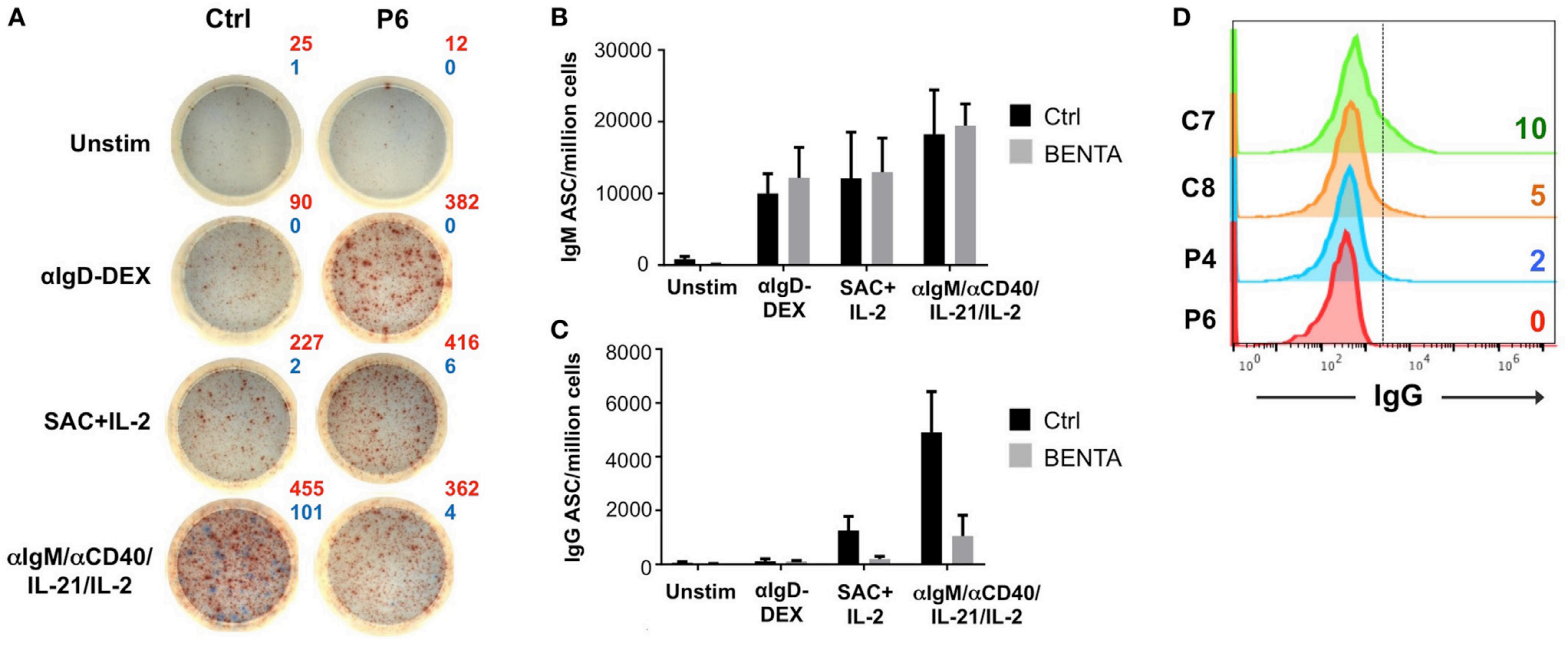
Decreased frequency of IgG antibody secreting cells (ASC) from *in vitro* stimulated B cell Expansion with NF-κB and T cell Anergy (BENTA) patient B cells. **(A)** Representative enzyme-linked immunosorbent spot (ELISPOT) data for control (left) and BENTA P6 (right) B cells following various activating stimuli. Red spots (IgM) and blue spots (IgG) indicate the number of antibody secreting cells as shown. Data are representative of six different experiments (*n* = 5 controls, four patients). Comparison of IgM **(B)** or IgG **(C)** ASC per million B cells (mean ± SEM) in control (black) and BENTA (gray) B cells analyzed in **(A)**. **(D)** Flow cytometric staining for surface IgG^+^ B cells for control (C7, C8) and BENTA patient (P4, P6) B cells stimulated with anti-IgM/anti-CD40/IL-21/IL-2 for 10 days.

We next measured the frequency of IgM and IgG antibody secreting B cells in BENTA patients using a dual-color ELISPOT assay (Figure 5A). Unstimulated control and BENTA B cells produced few IgM and IgG antibody secreting cells (ASC) *in vitro* as expected. Both αIgD-DEX and SAC + IL-2 stimulation generated variable but comparable numbers of IgM ASC from control and BENTA B cells (Figure 5B). Consistent with poor IgG secretion in culture supernatants, αIgD-DEX did not induce any IgG ASC cells in either group (data not shown). However, we observed a clear trend of reduced IgG ASC in BENTA B cell cultures stimulated with SAC + IL-2 and our anti-IgM/anti-CD40/IL-21/IL-2 cocktail (Figures 5A,C). Bar graphs shown in Figures 5B,C quantify the average number of control and BENTA IgM and IgG ASC per million B cells, respectively, across multiple experiments. Flow cytometric staining for surface IgG also revealed far fewer IgG^+^ cells in BENTA B cell cultures (Figure 5D). Collectively, these data highlight a reduced frequency of IgG^+^ ASC in stimulated BENTA B cell cultures, consistent with poor IgG production.

### Reduced Expression of Key PC Differentiation Factors in BENTA B Cells

**Figure 6:**
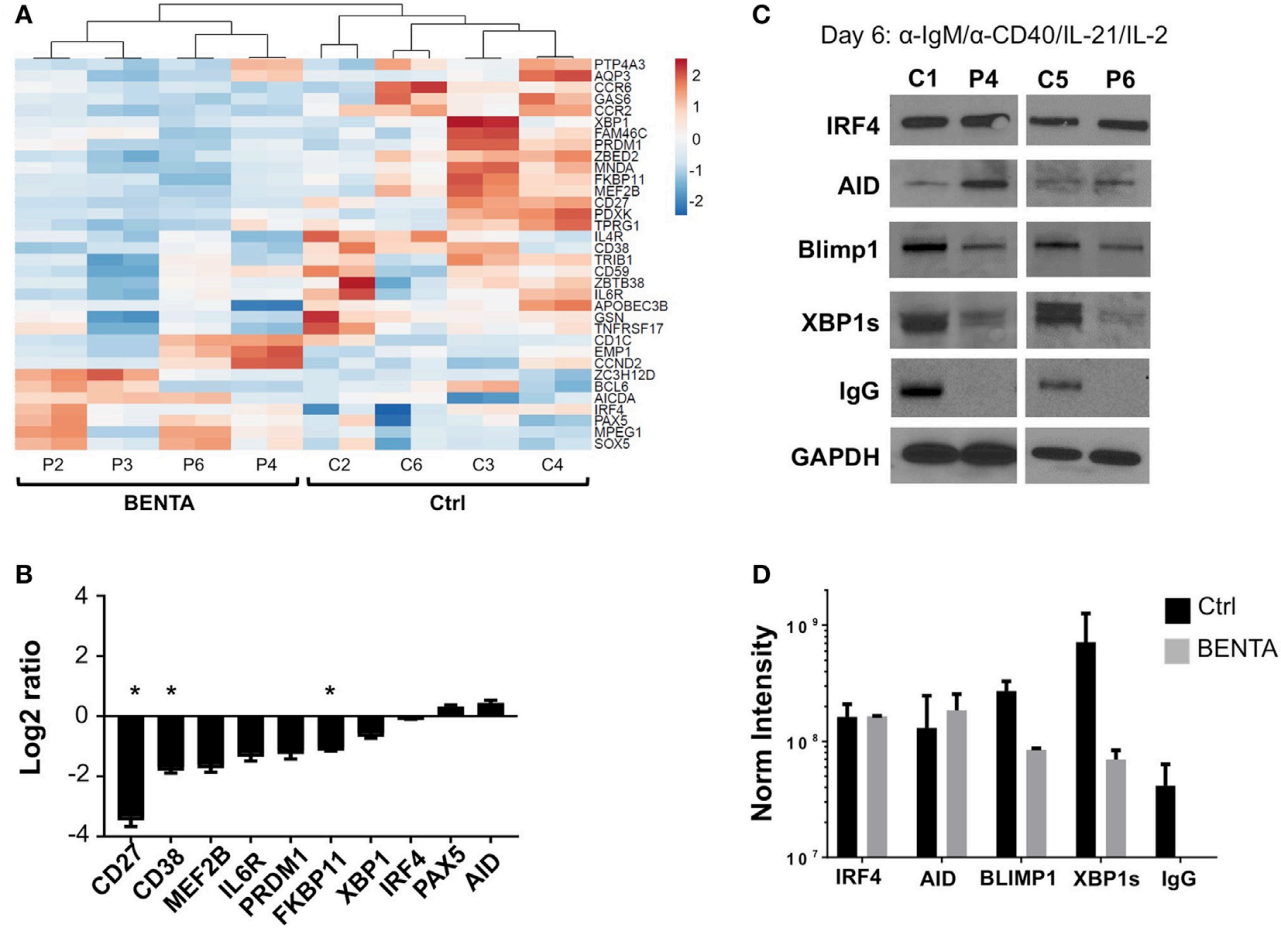
Lower expression of key plasma cell (PC) differentiation factors in activated B cell Expansion with NF-κB and T cell Anergy (BENTA) B cells. **(A)** Heatmap display of PC differentiation genes in activated control and BENTA RNA samples (day 4). Red color indicates high expression while blue color indicates low expression. Four different control and four different BENTA patient samples with two replicates were used for analysis. **(B)** Validation of gene expression comparing BENTA patients vs. controls (Log2 ratio, mean ± SEM) for select PC genes by qPCR. Data represent the average of relative normalized gene expression (based on housekeeping genes *HPRT2* and *RPL13*) from four different control and four different BENTA samples each with two replicates. Asterisks denote statistical significance (Student’s *t*-test with Welch correction, *p* < 0.05). **(C)** Representative immunoblots of B cell lysates prepared from activated naive control and BENTA B cells for the indicated proteins. Data are representative of three independent experiments (*n* = 4 controls, four BENTA patients). **(D)** Bar graph represents comparison of normalized densitometric intensity (mean ± SD) of key PC differentiation factors and IgG in control (black) and BENTA (gray) B cells shown in **(C)**. Protein expression was normalized to GAPDH expression.

To investigate possible molecular mechanisms behind defective IgG production and PC differentiation in BENTA patients, we performed RNA-Seq analysis for activated (anti-IgM/anti-CD40/IL-21/IL-2) control and BENTA patient B cells (day 4). Although we noted differential expression of multiple genes associated with B cell activation, survival, and metabolism, we focused our analysis on a signature of genes specifically tied to PC differentiation based on recent reports (26–28). As shown in Figure 6A, at least 33 PC-related genes were differentially expressed in control and BENTA groups each consisting of four different samples with two replicates. Ten B cell differentiation genes were selected for validation by qPCR. We confirmed that the expression of several PC-related genes trended lower in BENTA B cells compared to controls, including *CD27, CD38, MEF2B*, and *IL6R* (Figure 6B). Most importantly, BENTA B cells also expressed less *PRDM1*, which encodes BLIMP1, the master regulator of PC differentiation. Expression of the key B cell fate determinant gene *PAX5* was slightly higher in BENTA B cells compared to controls, although not statistically significant (Figures 6A,B). *PAX5* inhibits PC differentiation by repressing BLIMP1 expression in resting B cells and must be downregulated to allow the BLIMP1-dependent transcriptional program to proceed in developing PCs (29). In contrast, we noted comparable expression of activation-induced cytidine deaminase (*AID*, required for somatic hypermutation and class-switching) and interferon regulatory factor 4 (*IRF4*, an NF-κB-dependent transcription factor expressed early in PC differentiation) (Figure 6B).

We next asked whether reduced induction of essential PC differentiation factors was evident at the protein level by immunoblotting cell lysates made from day 6 activated control and BENTA B cells (anti-IgM/anti-CD40/IL-21/IL-2). Based on previous literature and our RNA-Seq results, we focused our analysis on a few key components in this process, including AID, IRF4, BLIMP1, and XBP1s (X-box binding protein 1 spliced variant, responsible for Ig production in long-lived PC) (27, 30, 31). The expression of these proteins was undetectable in unstimulated naive B cells (data not shown). Similar to our transcriptome analysis, we found that the expression of both AID and IRF4 was similar in both control and BENTA patient B cells following activation. In contrast, induction of BLIMP1 and XBP1s protein expression was reduced in BENTA patient cells compared to controls (Figure 6C). Spot densitometric quantification, normalized to housekeeping protein GAPDH expression for samples from both groups, supported this conclusion (Figure 6D). Interestingly, we also could not detect IgG heavy chain expression in BENTA patient B cell lysates, confirming that IgG production was impaired in BENTA B cells despite normal AID induction (Figures 6C,D). Altogether, these data suggest that PC differentiation is blocked in BENTA patient B cells due to a specific, intrinsic failure to induce essential late-stage factors including BLIMP1 and XBP1s after stimulation.

## Discussion

Our group previously discovered and described BENTA disease, a novel human congenital B cell lymphoproliferative disease caused by germline GOF mutations in *CARD11*. Thus far, five different GOF *CARD11* mutations have been discovered in 16 different patients (2, 17, 18) (unpublished data). These GOF mutations abrogate the requirement for antigen receptor engagement in CARD11 activation, rendering the scaffold protein in an “open” conformation that facilitates spontaneous oligomerization, enhanced interaction with BCL10 and MALT1, IKK activation, and constitutive NF-κB activation (2, 19). Elevated NF-κB activity likely plays a major role in the accumulation of immature and naive B cells in BENTA patients, manifesting as splenomegaly, lymphadenopathy, and B cell lymphocytosis early in life. However, clinical evaluation of most BENTA cases begins when affected children present with more frequent ear and sinopulmonary infections. Several lines of evidence suggest that BENTA disease includes an underlying immunodeficiency, including (a) low frequencies of circulating class-switched/memory B cells, (b) poor or transient antibody responses to selected vaccines (e.g., Pneumovax, Prevnar), and (c) mild T cell hyporesponsiveness *ex vivo*, which may relate to chronic viral infections (e.g., EBV) in some patients. Furthermore, autoantibodies are not readily detected in most patients despite profound B cell expansion.

In this study, we utilized purified B cells from healthy controls and our BENTA patient cohort to look for possible intrinsic defects in the B cell differentiation process for BENTA B cells after stimulation. Given the paucity of memory B cells in BENTA, we performed all experiments using purified naive B cells. Excess CD10^+^ immature transitional B cells seen in BENTA patients were eliminated during naive B cell isolation, as transitional cells quickly die in culture and could skew data interpretation (2).

Despite proliferating normally in response to polyclonal stimuli, we found that BENTA B cells could not differentiate into short-lived PC by day 6 in culture. Prolonging this assay for more than 6 days did not change the results, even though activated BENTA B cells displayed a survival advantage *in vitro* (Figures 2E,F). With the addition of extra cytokines including IL-6 and IFN-α, however, BENTA B cells did show hallmarks of short-lived PC differentiation by days 10–13 (e.g., IgD downregulation, CD38 induction), but failed to terminally differentiate into long-lived CD138^+^ PC. In fact, the addition of IL-6, IFN-α and IL-21 from the start of culture partially rescued short-lived PC differentiation in some BENTA patient B cells by day 6, but still failed to induce long-lived PC from BENTA B cells relative to controls (Figure S3 in Supplementary Material). Interestingly, the extent of “rescued” short-lived PC differentiation under these conditions seemed to correlate inversely with both B cell lymphocytosis and the *in vitro* survival advantage noted for individual patients (P2 < P4 < P6), although more studies and patients would be needed to verify this observation.

Both NF-κB and STAT3 signaling are required for proper PC differentiation; the integration of these signals ultimately govern key changes in the B cell transcription program. Our initial B cell stimulation cocktail included agonistic antibodies against the BCR and CD40, to activate both canonical and alternative NF-κB signaling pathways, respectively (32). NF-κB signaling normally triggers IRF4 expression in early phases of plasma cell commitment, initiating class switch recombination *via* AID induction in germinal center (GC) B cells. IL-21, a STAT3-activating cytokine normally supplied by T follicular helper cells *in vivo*, was also included in our cultures. Both CD40 and IL-21R signals are required to maximize BLIMP1 induction for optimal PC differentiation (33). Although IRF4 and AID expression were comparable in activated control and BENTA B cells, BLIMP1 induction was impaired in the latter. It is likely that hyperactive canonical NF-κB activity in BENTA B cells ultimately overwhelms or disrupts the balance of non-canonical NF-κB (i.e., CD40) and STAT3 signals required for optimal BLIMP1 expression and PC commitment. Even when cytokines such as IL-6 and IFN-α were added to boost STAT3 activation, long-lived PC differentiation could not be induced in BENTA B cell cultures. Reduced *IL6R* expression in BENTA B cells (Figures 6A,B) could contribute to decreased responsiveness. Failure to complete PC differentiation in BENTA B cells may also relate to sustained expression of *PAX5*, the critical governor of B cell identity that represses BLIMP1 expression in resting B cells. Little is known about the relationship between canonical NF-κB signaling and PAX5 regulation, although dysregulated non-canonical NF-κB (i.e., RelB) signaling is associated with an early block in B cell differentiation linked to reduced PAX5 expression (34). Nevertheless, our collective data suggest that constitutive CARD11 signaling impedes the transcriptional program associated with progression to PC fate, perhaps based on an imbalance in PAX5 versus BLIMP1 expression. Expression of *BCL6*, another important repressor of BLIMP1, was highly variable in controls and patients and did not show a significant difference in expression between groups (Figure 6A).

Consistent with poor PC differentiation, BENTA B cells also showed severely attenuated IgG production in response to both type 1-like (IL-10) and type 2-like (IL-4/IL-13) cytokine stimuli. ELISPOT analysis of BENTA B cell cultures confirmed a diminished frequency of IgG secreting cells in response to these stimuli, congruent with fewer cells expressing IgG on the cell surface. Reduced IgG production may relate to impaired upregulation of XBP1s, a transcription factor induced in a BLIMP1-dependent manner during late stages of terminal PC differentiation (35). XBP1s controls the unfolded protein response triggered by ER stress in highly secretory cells like PCs, ensuring the cell is equipped for sustained antibody synthesis (36). However, most BENTA patients show serum IgG levels in the lower end of normal range (1). This discrepancy could suggest that most of the IgG produced in BENTA patients might be oligoclonal and/or without affinity maturation, produced by short-lived PC developing outside normal GCs. Conversely, several BENTA patients often have low serum IgM levels even though our results suggest BENTA B cells can produce comparable or slightly increased amounts of IgM *in vitro*. This phenomenon could simply relate to enhanced survival of IgM secreting BENTA patient B cells *in vitro*, afforded by their relative resistance to apoptosis (17). Indeed, normal B cells become much more sensitive to apoptosis upon entering the GC reaction (37), which may not progress normally in BENTA patients (2).

Ultimately, *in vitro* culture systems for human GC or PC differentiation (particularly from naive B cells) are inefficient and incomplete, reflecting the difficulty in modeling the complex milieu of stromal and secreted signals that direct this process *in vivo*. Nevertheless, our findings illuminate a clear, intrinsic impairment in long-lived PC differentiation from BENTA patient B cells, based on specific defects in the PC transcriptional program that arise from overactive CARD11 signaling. A lack of long-lived PC for sustained production of affinity-matured antibodies may explain both repeated infections in BENTA patients, as well as the need for frequent boosting with certain vaccines to maintain protective titers. We are currently investigating direct causal links between constitutive NF-κB signaling, enhanced survival and intrinsic B cell differentiation defects, including evaluation of small molecule inhibitors that could simultaneously blunt excessive B cell accumulation and promote PC differentiation in BENTA patients. Moreover, BENTA-associated mutations may also perturb other CARD11-dependent signaling pathways such as mTORC1, deletion of which is known to abrogate GC formation and Ab production in peripheral B cells (4, 38).

## Ethics Statement

BENTA patient blood samples were obtained after written informed consent through protocols approved by the Institutional Review Boards (IRB) of the National Institutes of Health (NIAID protocol 06-I-0015) and USUHS (protocol PHA-75-2290).

## Author Contributions

SA designed, conducted, analyzed *in vitro* experiments, and wrote the article; BN assisted with experiments and bioinformatics analysis; NL, GS, and CD performed RNA-Seq and qPCR experiments and assisted with bioinformatics analysis; AS initially designed the project, supervised research, and edited the article.

## Conflict of Interest Statement

The authors declare that the research was conducted in the absence of any commercial or financial relationships that could be construed as a potential conflict of interest.
