# Supplementary material for: Intrinsic Plasma Cell Differentiation Defects in B Cell Expansion with NF-κB and T Cell Anergy Patient B Cells
Source: Front Immunol. 2017 Aug 2;8:913. doi: 10.3389/fimmu.2017.00913 (PMC5539167; doi:10.3389/fimmu.2017.00913)
Supplement: Supplementary file 1 [file presentation_1.pdf]

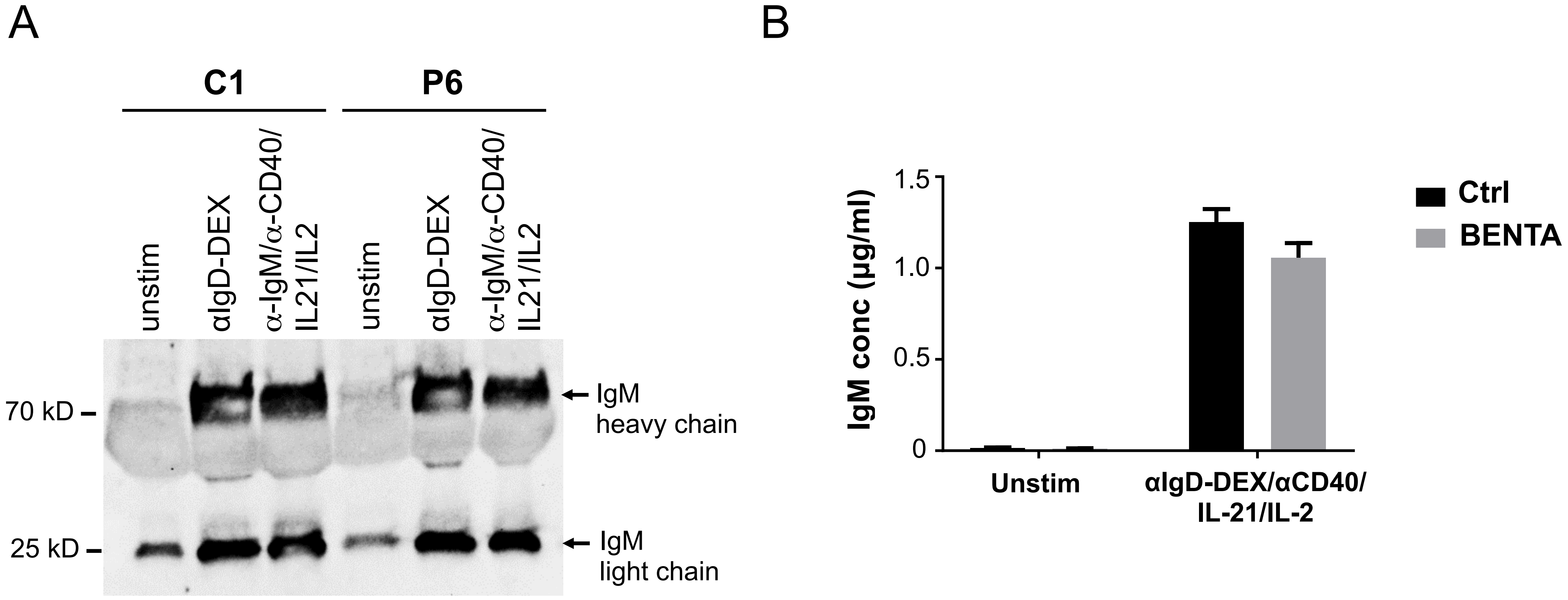

Supplemental Figure 1. T cell-dependent stimuli induce comparable IgM production from control and BENTA patient B cells. (A) Immunoblotting for IgM in cell supernatants collected from a healthy control (C1) and BENTA P6 naïve B cells following 10 days of culture -/+ activating stimuli shown. Arrows denote IgM heavy and light chain bands. Immunoblotting confirms IgM production under these conditions, where the agonistic anti-IgM Ab used for stimulation interferes with IgM detection by ELISA. (B) Supernatants from control (black) versus BENTA (grey) naïve B cells cultured with anti-IgD-dextran/ anti-CD40/IL-21/IL-2 were collected on day 10 and used for quantification of IgM. Data are mean ± SEM (n=2 controls, 2 patients).

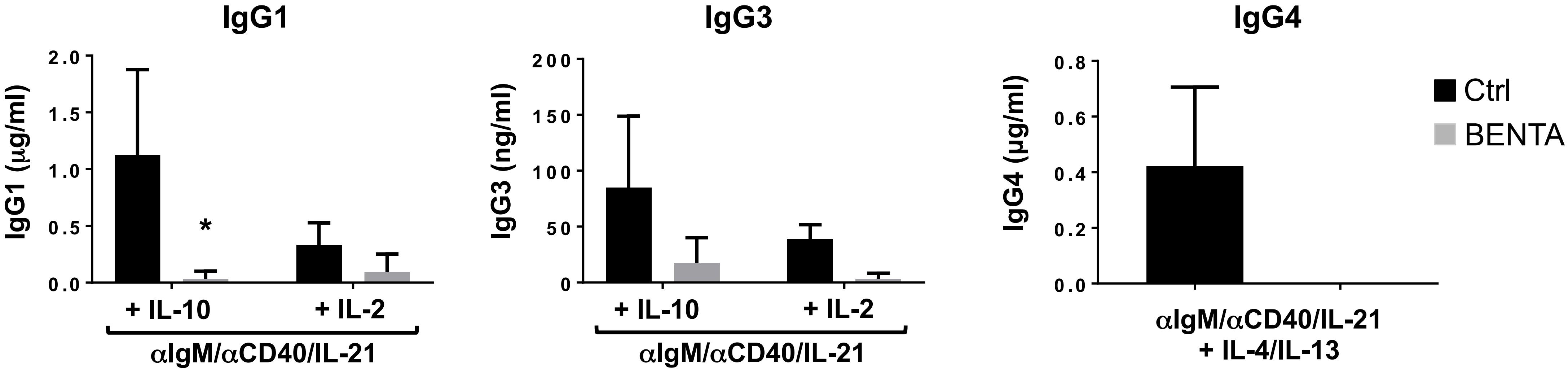

Supplemental Figure 2. Poor IgG subtype secretion in stimulated BENTA B cells. Supernatants from purified naïve B cells from healthy control (black) or BENTA (gray) B cells cultured with various activating stimuli were collected on day 10 and used for ELISA quantitation of IgG1 (+IL-10: n=4 controls, 3 patients; +IL-2 n=3 controls, 3 patients), IgG3 (+IL-10: n=4 controls, 4 patients; +IL-2 n=2 controls, 2 patients), and IgG4 antibodies (n=2 controls, 2 patients). Asterisk denotes significance (Student's t-test + with Welch correction, $p < 0.05$).

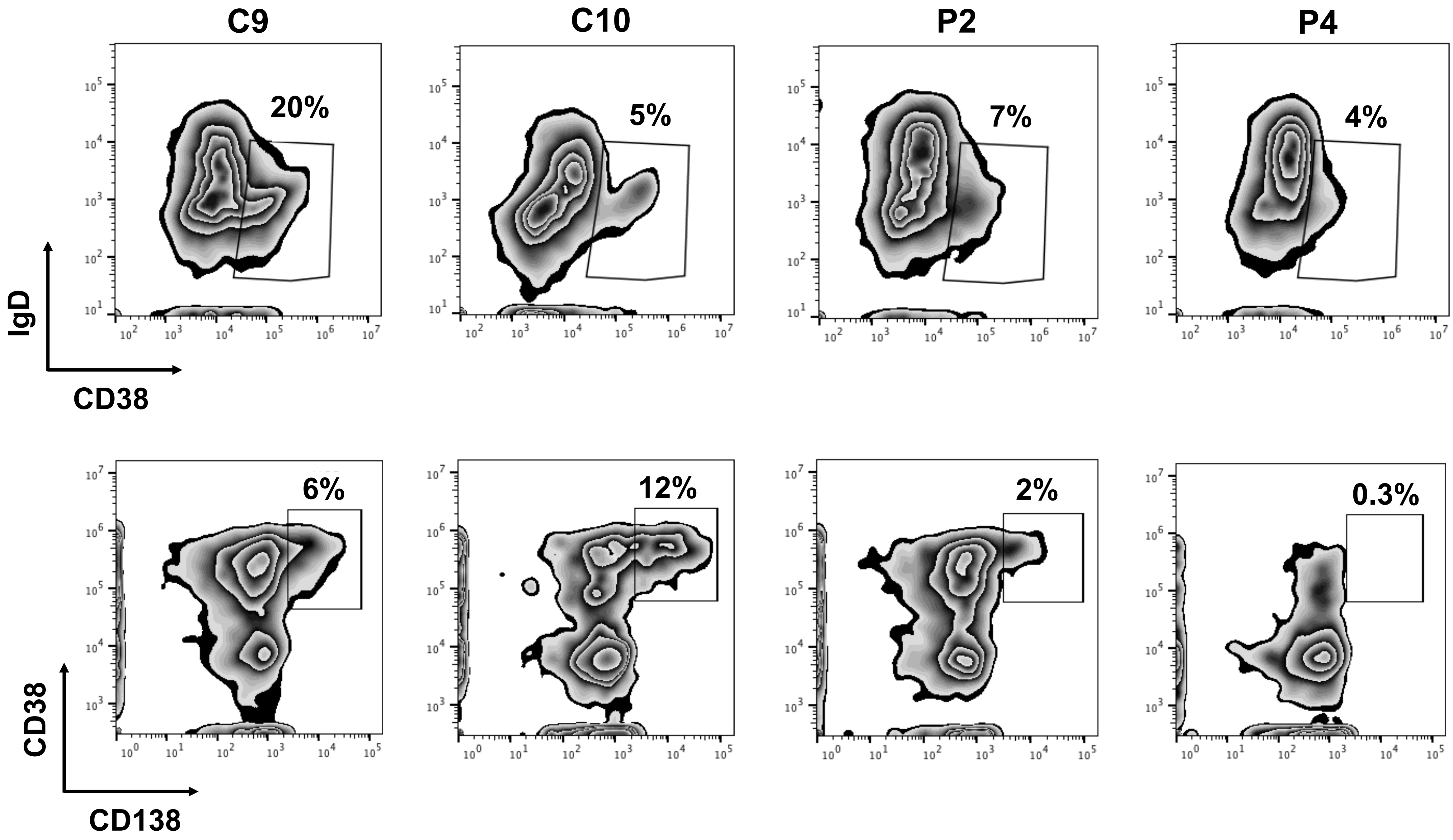

Supplemental Figure 3. Short-lived and long-lived PC differentiation *in vitro* in the continuous presence of IL-6 + IFN-α. Naïve B cells from healthy controls (C9, C10)) or BENTA patients (P4, P6) were stimulated with anti-IgM/anti-CD40/IL-21/IL-2 plus IL-6 + IFN-α from day 0. On days 6 and 13, respectively, the percentages of short-lived PCs (IgD^{lo} CD38^{hi} cells, top row) and of long-lived PCs (CD138^{hi} CD38^{hi} cells, bottom row) were identified by gating on viable cells based on forward/side scatter.
